# Acute decompensated heart failure in a non cardiology tertiary referral centre, Sarawak General Hospital (SGH-HF)

**DOI:** 10.1186/s12872-020-01793-7

**Published:** 2020-12-07

**Authors:** Hwei Sung Ling, Bui Khiong Chung, Pin Fen Chua, Kai Xin Gan, Wai Leng Ho, Elora Yoke Ling Ong, Cindy Hui San Kueh, Yie Ping Chin, Alan Yean Yip Fong

**Affiliations:** 1 Medical Department, Sarawak General Hospital (SGH), Kuching, Malaysia; 2 Faculty of Medicine and Health Sciences, University Malaysia Sarawak (UNIMAS), Jalan Datuk Mohammad Musa, 94300 Kota Samarahan, Sarawak Malaysia; 3 Clinical Research Centre, Sarawak General Hospital (CRC, SGH), Kuching, Malaysia; 4 Sarawak Heart Centre, Kota Samarahan, Malaysia

**Keywords:** Acute decompensated heart failure, Epidemiology, Sarawak, Southeast Asia, Malaysia

## Abstract

**Background:**

Data on clinical characteristics of acute decompensated heart failure (ADHF) in Malaysia especially in East Malaysia is lacking.

**Methods:**

This is a prospective observational study in Sarawak General Hospital, Medical Department, from October 2017 to September 2018. Patients with primary admission diagnosis of ADHF were recruited and followed up for 90 days. Data on patient’s characteristics, precipitating factors, medications and short-term clinical outcomes were recorded.

**Results:**

Majority of the patients were classified in lower socioeconomic group and the mean age was 59 years old. Hypertension, diabetes mellitus and dyslipidaemia were the common underlying comorbidities. Heart failure with ischemic aetiology was the commonest ADHF admission precipitating factor. 48.6% of patients were having preserved ejection fraction HF and the median NT-ProBNP level was 4230 pg/mL. Prescription rate of the evidence-based heart failure medication was low. The in-patient mortality and the average length of hospital stay were 7.5% and 5 days respectively. 43% of patients required either ICU care or advanced cardiopulmonary support. The 30-day, 90-day mortality and readmission rate were 13.1%, 11.2%, 16.8% and 14% respectively.

**Conclusion:**

Comparing with the HF data from West and Asia Pacific, the short-term mortality and readmission rate were high among the ADHF patients in our study cohort. Maladaptation to evidence-based HF prescription and the higher prevalence of cardiovascular risk factors in younger patients were among the possible issues to be addressed to improve the HF outcome in regions with similar socioeconomic background.

## Background

Heart failure (HF) is defined as complex clinical syndrome due to structural or functional impairment of heart, contributing to reduced cardiac output or raised intracardiac pressure [1]. In Asia, high prevalence of cardiovascular risk factors such as hypertension and diabetes are leading to more cardiovascular diseases such as ischemic related heart failure [2]. The present Asia Pacific registries are showing unsatisfactory HF outcome in this region [3].

In Malaysia, it was estimated 10% of admission to hospital was heart failure-related, and 25% of them readmitted within 30 days post-discharge.^1^ However, the reports on HF in Malaysia are scarce. Malaysian patients represented only small portion of data in the past regional clinical registries [3].

Sarawak, the largest state by area in Malaysia, ranked 4th in Malaysian population size, 5th in the gross domestic product, is located north in the land of Borneo and east in the country.^2^ Kuching is the capital city of Sarawak. Sarawak General Hospital (SGH) is a 1000-bed public hospital, providing tertiary care to Kuching and its regional townships which consist of at least 800,000 population. General medical department in SGH does not have in-house cardiology unit. There is a cardiology centre, Sarawak Heart Centre (SHC) located around 20 km away and clinical research centre (CRC) Kuching located in SGH. Hospitalisation, investigations and medications are provided at a subsidized rate for the patients in SGH. SGH is operating without an electronic database. Hence, all medical records are kept in written form.

Cardiology cases will be comanaged by SGH general physicians. Patients needed cardiology care will be transferred to SHC. Relevant cardiology test kits i.e., NT-ProBNP are currently made available for research purpose only.

Information on HF in this state is still lacking. Generalization of heart failure characteristics and outcomes using available data such as ADHERE-AP [3], is less suitable. Our population is unique, residing in a land full of challenging geographical terrain that affects the delivery of medical care.^3^ With only one cardiology centre in the entire state, little are known on the characteristics and outcomes of acute decompensated heart failure cases in the non-cardiology centres.

Hence, we want to address these knowledge gaps and discuss the possible reasons for the difference comparing with other major registries. This pioneer effort could provide information to improve HF management, steering the resource allocations of healthcare and contribute an idea for future studies. We believe these results on HF patients from non-urban areas may provide insights to the physicians from other developing countries with lower socioeconomic challenges.

## Objectives

Our observational study has several aims: first, to identify the clinical characteristics and demographics of acute decompensated heart failure (ADHF) patients in our centre; secondly, to illustrate the current management of patients hospitalised with ADHF; thirdly, to determine the clinical outcomes of patients with ADHF during hospitalisation, 1-month and 3-month post discharge; lastly, to compare the difference of clinical characteristics, managements and short term outcomes between patients with preserved and reduced ejection fraction.

## Methods

### Study design

This was a prospective cohort study.

### Study setting and data collection

Between September 2017 and August 2018, we recruited patients with a primary admission diagnosis of acute decompensated heart failure (ADHF) in Sarawak General Hospital.

Informed consent was obtained from each selected patient. A 3 mL venous blood was collected within 48 h of admission for NT-ProBNP test. The blood samples were stored in Ethylenediaminetetraacetic acid (EDTA) tube and sent to the clinical research centre within 1 h of blood taking. The overnight samples were kept in EDTA tubes under room temperature around 20–30 °C. Alere Triage ® NT-ProBNP point-of-care test kit and meter were used to determine the admission NT-ProBNP level. Patient’s baseline characteristics, comorbidities, admission investigation results, inpatient outcome, and the upon-discharge medications were recorded by reviewing the discharge notes. Discharged patients were followed up in SGH medical clinic or were contacted via phone calls to record their 30 and 90 days clinical outcomes.

### Study sampling, diagnosis definition, participants selection

We used convenient sampling. Recruitments were carried out from 8 am to 5 pm during weekdays. Study investigators were informed by the emergency department staff about the admissions of patient with a primary diagnosis of ADHF. Consecutiveness of recruitment was recommended but not checked with the admission log.

Heart failure diagnosis was made based on clinical assessment of presenting symptoms (i.e., Shortness of breath, orthopnoea, paroxysmal nocturnal dyspnoea, pedal oedema) and diagnostic investigations (i.e., chest x-ray, echocardiogram, N Terminal-pro Brain Natriuretic Peptide/NT-ProBNP). In our setting, general physicians commonly use Malaysian Heart Failure guideline as the reference.

Our study team pre-defined ischemic-related heart failure as any heart failure that was retrospectively proven by either a non-invasive test such as stress ECG, CT angiogram, or invasive coronary angiography. Other heart failure aetiology such as alcohol-related heart failure was based on the clinical judgement of treating physician. Heart failure with reduced ejection fraction was defined as ejection fraction of less than 40%.

Patients included in this study were 18 years and above. Patients had an admission creatinine clearance of at least 30 mL/min. Patients admitted after 5 pm until 8 am, during national holidays and over the weekend were not included due to limited access to echocardiographic and laboratory supports. Patients without discharge primary diagnosis of ADHF were o excluded. Patients uncontactable or lost to clinic follow up at 1 to 3 months after discharge were also excluded.

### Variables and measurements

#### Baseline characteristics

The baseline measurements recorded in this study were age, gender, ethnicity, origin, income status, education level, body mass index (BMI), background medical comorbidities such as hypertension, diabetes mellitus, dyslipidaemia, ischemic heart disease, valvular heart disease, atrial fibrillation, stroke, asthma, chronic obstructive pulmonary disease (COPD), New York Heart Association classification and past admission with heart failure within last 6 month. Heart rate, systolic blood pressure and diastolic blood pressure were recorded from admission and discharge clerking notes. Blood investigations including admission NT-proBNP level and electrocardiography test result were traced from the discharge notes.

#### Precipitating factors of heart failure and medications

Possible precipitators or aetiologies of heart failure were recorded as according to individual treating physician’s assessment. Each patient’s aetiology was traced from the discharge notes. The aetiologies mentioned include ischemic-related, hypertensive, atrial fibrillation, valvular heart disease and others. Heart failure medications during admission and prior to discharge were traced from the discharge notes.

#### Clinical outcome

We define inpatient outcome as all-cause mortality and the requirement of advanced cardiovascular support. Our near-term outcomes were 30-day all-cause mortality after discharge, 30-day hospital readmission, 90-day all-cause mortality, and 90-day readmission. The 30 and 90-day time frame were chosen due to the local practice of following up patients typically within 3 months after discharge.

#### Study size

This is the first report on the clinical characteristics of acute decompensated heart failure in our area. Hence, we did not prespecified a sample size but practiced convenient sampling.

### Statistical analysis

All statistical analysis was performed using SPSS 17.0 statistical software. Categorical variables are presented as percentages, while continuous variables are presented as mean and standard deviation if normally distributed, or as median, if not. Univariate analysis was performed on categorical variables by the Chi squared test and continuous variables by the independent t-test. Fischer exact test was used when expected count in a cell is less than 5. A *p *value of < 0.05 was considered statistically significant. Our approach to the missing data was to restrict the analysis to individuals with complete data on all variables required for a particular analysis. Percentages shown in the results were based on the number of non-missing values. No sensitivity analysis was performed.

### Ethics statement

This study was registered under the National Medical Research Register with approval from Malaysian Research Ethics Committee (NMRR-17-2314-37795). All participants provided informed consent in written form to participate in the study.

## Results

From September 2017 to October 2018, 117 patients were recruited. 10 patients who were not contactable during the follow up were excluded from this study. Table 1 summarizes the clinical characteristics, medications, precipitators and clinical outcomes of acute decompensated heart failure. The number of participants with complete data for each variable of interest were mentioned in the table of results. Table 2 showed the univariate comparison of variables between patients with reduced ejection fraction and preserved ejection fraction.

**Table 1:** (a) Baseline characteristics of ADHF patients comparing with other heart failure registries, (b) investigation findings, (c) precipitating factors and medications during admission and upon discharge, (d) clinical outcome

*n* (%) unless specified	*n* with available data in SGH-HF	SGH-HF	ADHERE-AP	EHFS II	ADHERE
(a)					
Age, years	107				
Mean, SD		59, 17.5	66	70	72
≥ 65 years, n (%)		50 (46.7)	–	–	–
Male gender	107	63 (58.9)	(57)	(61)	(48)
Ethnic					
Sarawak Ethnic	106	23 (22.5)			
Malay		48 (44.9)	–	–	–
Chinese		32 (29.9)			
Others		2 (1.9)			
Patient’s origin			–	–	–
Kuching	84	58 (54.2)			
Other districts		26 (24.3)			
Family income status					
Low	104	59 (55.1)	–	–	–
Medium		44 (41.1)			
High		1 (0.9)			
Education level					
Low	100	64 (59.8)	–	–	–
Medium		35 (37.1)			
High		1 (0.9)			
BMI, kg/m^2^, median {IQR}		25.7 {22.3–31.6}	–	26.8	–
Underweight		3 (2.8)	–	–	–
Normal		23 (21.5)	–	–	–
Overweight		37 (34.6)	–	–	–
Family history of coronary heart disease	104	5 (4.7)	–	–	–
Co-morbidity					
Hypertension	107	77 (72)	(64)	(62.5)	(73)
Diabetes mellitus	~	53 (49.5)	(45)	(32.8)	(44)
Dyslipidaemia	~	57 (53.3)	–	–	
Ischemic heart disease	~	33 (36.6)	(50)	(53.6)	(57)
Valvular heart disease	~	8 (7.5)	–	(34.4)	–
Atrial fibrillation	~	15 (14)	(24)	(38.7)	(31)
Stroke	~	8 (7.5)	(13)	(13.3)	–
Asthma	104	6 (5.6)	–	–	–
COPD	~	15 (14)	–	(19.3)	–
Admission with heart failure within last 6 months	104	23 (21.5)	–	(44.5)^#^	(33)
Heart rate admission/*discharge*, mean ± SD	105/*90*	103 ± 27/*83 ± 15*	–	95	–
Systolic BP admission/*discharge*, mean ± SD	104/*90*	146 ± 38/*123 ± 21*	–	135	144 ± 32.6
Diastolic BP admission/*discharge*, mean ± SD	104/*90*	86 ± 23/*71 ± 13*	–	80	–
NYHA					
I	106	19 (17.8)	–	–	(4)
II		27 (25.2)			(20)
III		43 (40.2)			(44)
IV		17 (15.9)			(32)
(b)					
Laboratory, mean, SD					
Serum urea, mmol/L	96	7.6, 4.6	–	–	–
Serum creatinine, mmol/L	97	119, 40	–	–	–
eGFR, mL/min	97	59, 20	133	–	–
Serum sodium, mmol/L	106	134.5, 5.4	–	–	–
Hemoglobin, g/dL	106	12.1, 2.5	–	–	–
NT-ProBNP, pg/mL, median {IQR}	63	4230 {2320–8160}	–	–	–
ECG, mean, SD					
Atrial fibrillation, n(%)	107	20 (18.7)	–	–	–
PR, ms	85	157, 32			
QRS, ms	104	97, 21			
> 120 ms	104	12 (11.2)			
QTc, ms	104	448, 59			
Echocardiography					
LVEF, %, median {IQR}	103	40 {29–58}	–	38 ± 15	34 ± 16
EF > 40%	103	52 (48.6)	(47)	(34.3)^	(4)
Moderate-severe TR	89	27 (25)	–	(29.9)	–
(c)					
Precipitating factors					
Ischemic HD	107	44 (41.1)	–	(30.2)	(57)
Hypertensive HD		8 (7.5)	–	–	–
Atrial fibrillation		6 (5.6)	–	(29.4)	–
Alcoholic HD		2 (1.9)	–	–	–
Valvular HD		6 (5.6)	–	(26.8)	–
Others		41 (38.3)	–	–	–
Pre-admission medications, n(%)					
ACEi/ARB	107	26 (24.3)	–	(64.3)	–
Frusemide	106	49 (45.8)	–	(71.2)	–
Spironolactone	~	13 (12.1)	–	(28.1)	–
Beta blocker	106	40 (37.4)	–	(86)	–
CCB	~	36 (33.6)	–	(17.8)	–
Digoxin	~	2 (1.9)	–	(26.6)	–
Lipid lowering agent	~	53 (49.5)	–	(28.4)	–
Ivabradine	~	4 (3.7)	–	–	–
Anticoagulation	~	13 (12.1)	–	(24)	–
Discharge medications, n(%)					
ACEi/ARB	96	30 (28.3)	(63)	(91)	(53)
Frusemide	96	76 (71)	–	(90)	(70)
Spironolactone	95	32 (29.9)	(31)	(47.5)	–
Beta blocker	96	51 (47.7)	(41)	(61.4)	(48)
CCB	96	24 (22.4)	–	(14.6)	–
Digoxin	96	4 (3.7)	(34)	(31)	(28)
Lipid lowering agent	96	56 (52.3)	–	(41.8)	–
Ivabradine	96	7 (6.5)	–	–	–
Anticoagulation	95	15 (14)	(18)	(33.1)	–
(d)					
In hospital outcome, n(%)					
Mortality	107	8 (7.5)	(4.8)	239 (6.7)	(4)
Discharge home	~	96 (89.7)	–	–	–
DAMA/transfer to other hospital	~	3 (2.8)	–	–	–
Length of stay, median {IQR}, days	106	5 {4–9}	6	9 {6–14}	4
Requirement of advanced cardiopulmonary support ± ICU care	105	46 (43)	–	(51)	–
30-day outcome, n(%)					
All-cause mortality	92	14 (13.1)	–	–	–
Readmission	84	12 (11.2)			
90-day outcome, n(%)					
All-cause mortality	92	18 (16.8)	–	–	–
Readmission	81	15 (14)			

The ‘admission’ vs ‘discharge’ values are in italics

*SD* standard deviation, *IQR* interquartile range, *ADHERE AP* Acute Decompensated Heart Failure Registry International Asia Pacific, *EHFS II* EuroHeart Failure Survey II, *ADHERE* Acute Decompensated Heart Failure Registry, *COPD* Chronic obstructive Pulmonary Disease, *BP* blood pressure, *NYHA* New York heart association, *eGFR* estimated Glomerular filtration rate, *LVEF* left ventricular ejection fraction, *TR* tricuspid regurgitation, *HD* heart disease, *ACEi* angiotensin converting enzyme inhibitor, *DAMA* discharge against medical advice, *ICU* intensive care unit, ^ EF more than 45%, ^#^admission with HF last 12 months, ~ similar value with previous number

**Table 2:** (a) Comparison of baseline characteristics in ADHF patients according to Ejection Fraction (EF), (b) investigation results of ADHF patients in SGH-HF according to EF, (c) precipitating factors, admission and discharge medications of ADHF patients in SGH-HF according to EF, (d) clinical outcomes of ADHF patients in SGH-HF according to EF

n (%) unless specified	Preserved EF	Reduced EF	*p* value
	n = 52	n = 55	
(a)			
Age, years			
Mean, SD	63, 17.2	55, 17.3	0.024*
Gender			
Male	25 (48.1)	36 (70.6)	0.02*
Female	27 (51.9)	15 (29.4)	
Ethnic			
Sarawak ethnic	9 (17.3)	12 (24.5)	0.558
Malay	24 (46.2)	24 (49.0)	
Chinese	18 (34.6)	13 (26.5)	
Others	1 (1.9)	–	
BMI, kg/m^2^, median {IQR}	26.5 {22.2–32}	25.7 {22.4–31.8}	0.605
Underweight	1 (4.0)	2 (5.4)	
Normal	9 (36.0)	13 (35.1)	1.0
Overweight	15 (60)	22 (59.5)	
Co-morbidity			
Hypertension	43 (82.7)	32 (62.7)	0.023*
Diabetes mellitus	31 (59.6)	20 (45)	0.038*
Dyslipidaemia	34 (65.4)	22 (43.1)	0.023*
Ischemic heart disease	17 (32.7)	18 (35.3)	0.78
Valvular heart disease	5 (9.6)	3 (5.9)	0.715
Atrial fibrillation	10 (19.2)	5 (9.8)	0.175
Stroke	3 (5.8)	4 (7.8)	0.715
Asthma	3 (5.9)	3 (6.1)	1.0
COPD	7 (13.5)	8 (15.7)	0.749
Heart rate admission/*discharge*, mean ± SD	101 ± 26/*84 ± 16*	103 ± 25/*83 ± 16*	0.687/*0.874*
Systolic BP admission/*discharge*, mean ± SD	151 ± 35/*128 ± 24*	140 ± 38/*118 ± 18*	0.159/0.027*
Diastolic BP admission/*discharge*, mean ± SD	84 ± 23/*72 ± 13*	89 ± 23/*71 ± 14*	0.313/*0.718*
NYHA			
I	12 (23)	6 (12)	0.154
II	11 (21.2)	16 (32)	
III	23 (44.2)	17 (34)	
IV	6 (11.5)	11 (22)	
(b)			
Laboratory, mean, SD			
Serum urea, mmol/L	7.7, 5.2	7.6, 4.2	0.926
Serum creatinine, mmol/L	124, 45	116, 34.7	0.366
eGFR, mL/min	54, 18	61, 20.1	0.084
Serum sodium, mmol/L	134.5, 6.1	135, 4.8	0.463
Hemoglobin, g/dL	11.5, 2.9	12.8, 2.1	0.012*
NT-ProBNP, pg/mL, mean {IQR}	5616 {1400–8160}	8760 {2695–10,015}	0.123
ECG, mean, SD			
Atrial fibrillation, n(%)	9 (17.3)	9 (17.6)	–
PR, ms	162, 36	154, 27	0.258
QRS, ms	98, 25	97, 16	0.99
QTc, ms	445, 69	455, 45	0.425
(c)			
Precipitating factors, n(%)			
Ischemic HD	9 (17.3)	10 (19.6)	0.432
Hypertensive HD	4 (7.7)	4 (7.8)	
Atrial fibrillation	4 (7.7)	1 (2)	
Alcoholic HD	1 (1.9)	1 (2)	
Valvular HD	5 (9.6)	1 (2)	
Others	29 (55.7)	34 (66.7)	
Pre-admission medications, n(%)			
ACEi/ARB	10 (19.2)	16 (31.4)	0.156
Frusemide	17 (32.7)	30 (58.8)	0.017*
Spironolactone	4 (7.7)	9 (17.6)	0.149
Beta blocker	18 (34.6)	21 (41.2)	0.541
CCB	23 (44.2)	12 (23.5)	0.037
Digoxin	1 (1.9)	1 (2)	1.0
Lipid lowering agent	26 (50)	26 (51)	1.0
Ivabradine	0	4 (7.8)	0.057
Anticoagulation	10 (19.2)	3 (5.9)	0.072
Discharge medications, n(%)			
ACEi/ARB	12 (24.5)	18 (40)	0.091
Frusemide	40 (81.6)	34 (77.3)	0.603
Spironolactone	12 (25)	18 (45.9)	0.104
Beta blocker	24 (49.0)	26 (59.1)	0.329
CCB	18 (36.7)	6 (13.6)	0.011*
Digoxin	2 (4.2)	1 (2.3)	1.0
Lipid lowering agent	27 (55.0)	27 (61.4)	0.541
Ivabradine	0	6 (13.6)	0.009*
Anticoagulation	9 (18.8)	5 (11.4)	0.324
(d)			
In hospital outcome, n(%)			
Mortality	2 (3.9)	6 (12.0)	0.16
Length of stay, mean {IQR}, days	7 {4–9}	7 {4–9}	0.813
Requirement of advanced cardiopulmonary support ± ICU care	26 (52)	17 (33.3)	0.058
30-day Outcome, n(%)			
All-cause mortality	5 (11.1)	9 (20.5)	0.226
Readmission	3 (7.0)	8 (21.0)	0.065
90-day outcome, n(%)			
All-cause mortality	6 (13.3)	12 (27.3)	0.102
Readmission	4 (9.5)	10 (27.8)	0.036*

The ‘admission’ vs ‘discharge’ values are in italics

*SD* standard deviation, *IQR* interquartile range, *COPD* Chronic obstructive Pulmonary Disease, *BP* blood pressure, *NYHA* New York heart association, *eGFR* estimated Glomerular filtration rate, *HD* heart disease, *ACEi* angiotensin converting enzyme inhibitor, *ICU* intensive care unit, ^EF more than 45%, ~ similar value with previous number

### Baseline characteristics of ADHF patients

The mean age of the cohort was 59 years old. Half of the cohort were classified in the geriatric group. 58.9% were males, and 22.5% of patients were from Sarawak ethnic groups. 24.3% of the cohort reside outside Kuching district, showing the burden of disease in non-tertiary district medical centres. 55% patients were classified in the lower-income group and 59% had attained low-level of education.

34% of patients were obese, with median BMI at 25.7. The common co-morbidities observed among the cohort include hypertension (72%), dyslipidaemia (53%), diabetes mellitus (49%) and ischemic heart disease (36.6%).

During admission, the mean heart rate, systolic and diastolic blood pressure was 103, 146 and 86 respectively. The predominant presenting NYHA class was III.

### Investigation findings during admission

Table 1b showed the laboratory, ECG and echocardiographic results. The mean eGFR in the cohort was 59 mL/min and the mean serum urea level was 7.6 mmol/L. The cohort recorded mean serum sodium of 134.5 mmol/L and 12.1 g/dL for the haemoglobin level. The median NT-ProBNP was 4230 pg/mL.

Most patients were in sinus rhythm (81.3%). A smaller number of the cohort had a QRS interval of > 120 ms (11.2%). The mean QTc duration was near to high borderline normal value, 448 ms ± 59.

The median of LVEF was 40%. Near to half of the cohort (48%) were classified under preserved ejection fraction EF heart failure. 25% patients had moderate to severe echocardiographic findings of tricuspid regurgitation.

### Precipitating factors for ADHF admission and medication prescribed

Table 1c displayed the precipitating factors, pre-admission and discharge medications for the cohort. The most prevalent precipitating factors of heart failure was ischemic heart disease (41.1%). Forty-one cases (38.3%) of heart failure were not specified for the possible cause. In combination, 20.6% of the cohort had hypertensive, arrhythmia, valvular and alcoholic related cardiomyopathy.

There was a general increase in the prescription of HF medications during hospitalisation, except calcium channel blocker. Patients who received spironolactone doubled in number from 13 to 32 patients. Among the pre-admission medications, lipid-lowering agents (49.5%) and frusemide (45.8%) appeared most often in the patient’s drug lists. The three most common anti-hypertensive drugs prescribed include beta-blocker at 37.4%, calcium channel blocker at 33.6% and ACE inhibitor/ARB at 23.3%. Upon discharge, frusemide (71%) and lipid-lowering agents (52.3%) remained the most common medications prescribed. Other medications saw increment in prescription: beta-blocker (47.7%), spironolactone (29.9%), ACE inhibitor/ARB (28.3%), and ivabradine (6.5%).

### In hospital, 30 and 90-day outcomes

Table 1d outlined the in-hospital outcome, and near term follow up data. A total of 8 (7.5%) in-hospital death were recorded. 43% of the patients required advance cardiopulmonary support or ICU treatment during the entire hospital stay. Patients stayed around 5 days in the hospital. 13% and 17% of the cohort recorded mortality during 30 and 90 days from admission. Readmissions were seen at the rate of 11% and 14% in 30 to 90 days from the index event.

### Comparison between preserved EF and reduced EF heart failure

Table 2 showed the difference of baseline characteristics, investigations, precipitating factors of heart failure, medications and clinical outcomes between patients with preserved and reduced ejection fraction heart failure. Patients in the reduced EF group were significantly more likely to be male and younger. Patients with preserved EF had more hypertension, type 2 diabetes and dyslipidaemia as their background illness. They were also found to be more likely to have lower haemoglobin count. Both groups had similar presenting vital signs and NYHA classification. Patients in the reduced EF group recorded higher NT-ProBNP level during their admission. There were no significant differences between 2 groups in the factors leading to heart failure. A lower rate of prescription of evidence-based medication were seen among patients with preserved EF. The hospital stay duration was similar across both groups. More deaths and readmission were recorded at 30-day and 90-day among patients with reduced EF. 50% of patients in the preserved EF group received advanced tertiary care during admission.

## Discussion

To our best knowledge, our study first reported acute decompensated heart failure characteristics and outcome data for patients in Sarawak. We compared and discussed our findings with the previous Malaysian data derived from a single centre study and other region’s heart failure registries. Although our study was limited with small number of participants, our study’s geographical area resembles places with lower socioeconomic background without specialised cardiology facilities. This comparison can be used as a reference to customise and improve the future heart failure care in places with similar background characteristics.

Our study participant numbers were not high due to several reasons. HF cases presented to the surrounding medical facilities were not included. Exclusion of patients with creatinine clearance < 30 mL/min and patients without primary admission diagnosis of HF had markedly reduce the recruitment rate. Difficulty to enrol patients during non-office hours was another hindrance to the recruitment. Another possible reason of low recruitment rate was due to the need of patient to be consented for the study. Proper consent was needed in this study as our NT-ProBNP test kits were currently meant for research purpose only. We envision the NT-proBNP data generated from this observational study can be the first step to allow more generalized use of this important tool to aid in the management of HF in our centre in the future.

The patients in SGH-HF were younger (mean age of 59) compared with previous Malaysian 63, Asia Pacific 66 and Western countries 70 data [3–5]. Two possible reasons are highlighted here: the high prevalence of cardiovascular risk factors and potential suboptimal health awareness affecting lifestyle and diet. Malaysia had recorded high number and younger patients (47% of the population at the age 30 years and above) with at least one cardiovascular risk factors.^4^ Sarawak 0.737 has a lower HDI in comparison to average Malaysia HDI 0.853, Singapore 0.944, and United Kingdom 0.922. Lower human development index (HDI) has a significant correlation with heart failure admission among the younger age group [6].This could explain the poor health awareness among our patients who are young with low socioeconomic and education background, leading to higher rate of cardiovascular diseases. Of note, our study observed a large group of geriatric cohorts (46.7%) in tandem with the increasing geriatric population size in Malaysia and Europe. Management of HF in geriatric age group expects challenges due to poorly tolerated therapy and multiple comorbidities [7].

Epidemiological transition and higher prevalence of CV risk factors in developing countries are the plausible causes to higher rate of ischemic related heart failure in our population [8, 9]. Younger age group in our study also explains the lower rate of atrial fibrillation, which agrees with the recent Malaysian AF data [10]. Patients in our study presented with more severe symptoms compare to the past Malaysia data. This suggests that accessibility to healthcare facilities could be a factor to consider [11].

NT-ProBNP level in our cohort was much lower (median: 4230 pg/mL) as compared to ASCEND-HF trial 5773 pg/mL, PRIMA II trial 6350 pg/mL and Taiwan 11,530 pg/mL. Younger age population, better renal function and more preserved ejection fraction HF were among the postulated reasons among our patient, [12–14].

The evidence-based HF medication prescription rate in SGH-HF cohort was lower than previous Malaysian, Asia Pacific and Western countries data. In Asia, regional differences were seen and developing countries were less adherence to the guidelines [15]. Drug tolerability in Asian and geriatric population are among the reason heavily debated [16]. Treating physician are hesitant to commence ACE inhibitor as many of our population were older with renal impairment and reside far in rural areas with limited medical facilities [17]. Limitation of in-house specialized cardiology services is another factor to poor adherence to the guideline directed therapy. Our study population tend to have multiple comorbidities. Hence, polypharmacy may hinder physician to start all HF medication at once during discharge.

The higher in-hospital mortality 7.5% in our study compared to Peninsular Malaysia 5.2%, Asia 4.8% and Western 4.0–6.7% data is alarming [11, 18]. The 1-month and 3-month mortality rate was higher too [18]. We postulated that our study population may have presented to the hospital at later stages of illness, reflected by their higher presentation NYHA status. The poor adherence to evidence-based medication, multiple comorbidities, lower literacy and presence of more geriatric patients are also hypothesized to cause higher mortality. Late or severe HF presentation in our cohort may translated into the frequent usage of advanced cardiopulmonary or ICU care.

The hospital stay duration was short for our cohort. We attribute this to the congested bed occupancy situation in our centre. Medical department in Sarawak General Hospital had recorded high bed occupancy rate in the recent 2017 report (96.7%).^5^ A contrary hypothesis to the short hospital stay was the administration of early advanced cardio-pulmonary care which had promoted faster recovery among the patients [19]. It is unfortunate to not document cardio-rehabilitation regimen in our study that may affect the length of stay, such as in Japan [20]. Our lower observed readmission rate could be due to limited centralized patient database to capture the recurrent admission record in the rural hospitals. The lower socioeconomic status may also limit the accessibility of patients to the medical facilities [21].

The proportion of patients with preserved ejection fraction heart failure was near to the findings of Asia-Pacific and US study. High prevalence of cardiovascular risk factors including ischemic heart disease in our cohort maybe the factor leading to more diastolic and systolic dysfunction [22, 23]. The clear reason was the association of cardiovascular risk factor with the atherosclerotic coronary diseases [4, 24]. The systolic blood pressure during discharge was significantly lower among reduced EF cohort. This was most likely due to more prescription of anti-failure medications in the group. The lower rate of ACE inhibitor, beta-blockers and spironolactone prescription among preserved EF patients was likely driven by lack of guideline-directed therapy.

Generally, the comparisons of clinical outcomes between preserved and reduced EF patients yielded inconsistent results [25]. One of the reasons was the variable study design and definitions of preserved EF. In our cohort, more patients with reduced EF were readmitted for heart failure within 90 days from the discharge. Lower EF was usually associated with poorer long-term prognosis [26, 27]. High clinical events among our study patients with preserved EF may be due to their older age and the presence of multiple comorbidities. Our team concluded both groups of HF patients required similar amount of attention as no statistical difference was observed in terms of their short-term clinical outcome.

Our study revealed high mortality and readmission rate among heart failure patients in East Malaysia which was never describe before. This data could increase the awareness of many physicians about the impact of suboptimal HF treatment. We discussed a few learning points that are applicable to regions with similar socioeconomic background, with the purpose to improve the quality of heart failure care. Our patients are more socioeconomically disadvantaged and hence are less accessible to the tertiary care. Suburban and rural healthcare staffs should be trained to ensure the continuity of heart failure care after the discharge. This could address the lower rate of ACE inhibitor or ARB prescription which requires strict near-term creatinine monitoring after the initiation of therapy. HF case detection rate can be improved by training the healthcare staff to screen for HF symptoms among patients who are attending the local general clinics for their comorbidities treatment such as hypertension. To improve case detection, NT-ProBNP should be make more accessible in all healthcare centres. Our study highlighted the need of joint effort between all healthcare facilities to contribute into the local heart failure registry in less developed area. Outcome findings from the registry could serve as evidences for hospital and clinic administrators to properly allocate resources to improve the HF services in the future.

Our preliminary data has several limitations. The results cannot be generalized to the entire Sarawak population, limited by the small sample size and single centre sampling. Selection bias was presence as patients from outpatient clinics that may differ in ADHF presentation were excluded. Patients from rural areas were likely underrepresented due to the small study population. Our study also excluded the common disease of chronic kidney disease which is often associated with pathophysiology and complication of ADHF [28]. Furthermore, we neglected the outpatient care details which had prevented us from concluding its impact on the outpatient or longer-term clinical prognosis.

## Conclusion

In conclusion, this is the first observational prospective data on ADHF in the land of Borneo, showing notable differences with Western countries and echoed certain similarities with data from other Asian countries. Our findings represented some similarities with the previous data from countries with lower socioeconomic background. Younger, lower socio-economic-education status and high prevalence of cardiovascular risk factors were among notable findings relevant in public health perspectives. Suboptimal adherence rate to the evidence-based HF medication prescription should be the next focus of improvement in our region for better mortality and readmission outcomes. Local HF registry with more comprehensive outpatient and inpatient HF data should be initiated to fill in the knowledge gap on HF population in our region.

## Data Availability

The datasets used and analysed during the current study are available from the corresponding author on reasonable request.

## Footnotes

1 Ministry of Health Malaysia, *Management of Heart Failure* 2014, 3rd ed.

2 Department of Statistics Malaysia, *Statistics by themes in Malaysia* 2019.

3 Abdullah NHB, *Sarawak Healthcare System, Director-General of Health’s office Malaysia, 2014.*

4 Ministry of Health Malaysia, *Clinical Practice Guideline on Primary and Secondary Cardiovascular Disease*, 2017, 1st ed.

5 Sarawak General Hospital, *Annual Report SGH*, 2017.
